# *microRNA*-*660* Enhances Cisplatin Sensitivity via Decreasing SATB2 Expression in Lung Adenocarcinoma

**DOI:** 10.3390/genes14040911

**Published:** 2023-04-14

**Authors:** Ziyao Wang, Lingxuan Zhou, Bisong Chen, Xu Li, Qiuyi Zou, Wei Xu, Li Fang, Anbang Wu, Zheng Li, Yuejun Chen

**Affiliations:** 1 Department of Thoracic Surgery, The Affiliated Cancer Hospital of Xiangya School of Medicine, Central South University/Hunan Cancer Hospital, Changsha 410006, China; 2 Department of Pathophysiology, Institute of Basic Medicine, Hebei Medical University, 361 Zhongshan East Road, Shijiazhuang 050017, China; 3 NHC Key Laboratory of Carcinogenesis, The Affiliated Cancer Hospital of Xiangya School of Medicine, Central South University/Hunan Cancer Hospital, Changsha 410006, China

**Keywords:** *miR*-*660*, SATB2, cisplatin sensitivity, lung adenocarcinoma

## Abstract

Increasing evidence suggests that microRNAs’ (miRNAs) abnormal expression is one of the main factors of chemotherapy resistance in various cancers. However, the role of miRNAs in lung adenocarcinoma (LUAD) resistance to cisplatin is still unclear. In this study, we analyzed a microarray dataset to investigate miRNAs related to cisplatin resistance in LUAD. The expression of miRNAs in LUAD tissues and cell lines was detected using real-time quantitative polymerase chain reaction (RT-qPCR). Special AT-Rich Sequence-Binding Protein 2 (SATB2) in LUAD cell lines was detected using RT-qPCR and Western blot. Cell proliferation was measured by CCK8 and colony formation assays, while cell cycle and apoptosis were measured by flow cytometry. A dual-luciferase reporter assay was performed to confirm that SATB2 is a target gene of *microRNA*-*660* (*miR*-*660*). We showed that the expression of *miR*-*660* was not only decreased in LUAD cells and tissues but also further decreased in the cisplatin-resistant A549 cell line. The overexpression of *miR*-*660* increased cisplatin sensitivity in LUAD cells. In addition, we identified SATB2 as a direct target gene of *miR*-*660*. We also revealed that *miR*-*660* increased cisplatin sensitivity in LUAD cells via targeting SATB2. In conclusion, *miR*-*660*/SATB2 axis is a key regulator of cisplatin resistance in LUAD.

## 1. Introduction

Lung cancer is the leading cause of cancer-related deaths globally, leading to over 20% of cancer deaths [1]. Lung adenocarcinoma (LUAD) is a pathological subtype of lung cancer [2]. LUAD patients who have no indications for surgical interventions are often treated with medical therapy. In the past two decades, numerous biomarkers of LUAD have been found, resulting in targeted therapy and immunotherapy development. For example, gefitinib for EGFR mutation, clothotinib for ALK-EML4 fusion, and pembrolizumab for PD-1 molecular have greatly extended the survival of LUAD patients [3,4]. Despite the rapid development of multiple therapies, platinum-based chemotherapy remains the main therapy regimen for LUAD patients. Cisplatin, a typical platinum drug, is capable of killing tumor cells. Dual-drug chemotherapy based on cisplatin is not only the first-line treatment of LUAD but also the preferred drug of chemotherapy combined with targeted therapy or immunotherapy [5]. However, LUAD cells may develop resistance to cisplatin, which ultimately results in treatment failure [6]. Therefore, it is necessary to find a way to overcome cisplatin resistance in LUAD.

MicroRNAs (miRNAs), a kind of short (20–24 nucleotides) non-coding RNA, can regulate gene expression post-transcriptionally by repressing translation or degrading targeted messenger RNAs (mRNAs) [7,8]. MiRNA dysregulation affects a spectrum of physiological and pathological processes in various cancers, including proliferation, metastasis, and drug resistance [9]. It has been reported that *miR*-*205* can enhance chemotherapy resistance in non-small cell lung cancer via targeting the phosphatase and tensin homolog [10]. Chen et al. showed that *miR*-*181a* overexpression degrades protein kinase C delta, thereby inducing cisplatin resistance in human cervical squamous cell carcinoma [11]. However, the role of miRNAs in regulating cisplatin sensitivity in LUAD is still not fully clear.

In this study, we screened potential miRNAs related to cisplatin resistance in LUAD by identifying differentially expressed miRNAs in cisplatin-resistant and cisplatin-sensitive LUAD cell lines. We found that the expression of *microRNA*-*660* (*miR*-*660)* was significantly decreased in the cisplatin-resistant LUAD cell line. *MiR*-*660* overexpression reduced cisplatin resistance in LUAD. Moreover, we revealed that *miR*-*660* targeted Special AT-Rich Sequence-Binding Protein 2 (SATB2), thereby impairing cisplatin resistance in LUAD. This study suggests that *miR*-*660* and SATB2 might potentially be new therapy targets for reversing cisplatin resistance in LUAD.

## 2. Materials and Methods

### 2.1. Bioinformatics Analysis

We screened LUAD cisplatin sensitive/resistance microarray datasets in the Gene Expression Omnibus database [12]. Two datasets, GSE84200 [13] and GSE43493 [14], were selected that contained the miRNA and mRNA expression profiling data, respectively. The R language program (version 3.5.0) was used to analyze differentially expressed genes (Student’s *t*-test, log2(fold-change) ≥ 1 and *p* < 0.05), and analysis results were visualized as heatmap and volcano plots using the pheatmap package (https://cran.r-project.org/web/packages/pheatmap/index.html/, accessed on 9 April 2023, v1.0.12) and the ggplot2 package [15]. The dbDEMC (https://www.biosino.org/dbDEMC/index, accessed on 9 April 2023, v3.0) [16] and starBase (https://starbase.sysu.edu.cn/, accessed on 9 April 2023, v3.0) [17] databases were used to analyze the expression and prognosis of miRNAs and mRNAs in LUAD. The Genomics of Drug Sensitivity in Cancer (GDSC) (https://www.cancerrxgene.org/, accessed on 9 April 2023, v8.3) [18] database was used to obtain the cisplatin half-maximal inhibitory concentration (IC_50_) values of LUAD cell lines. The miRWalk (http://mirwalk.umm.uni-heidelberg.de/, accessed on 9 April 2023, v6.0) [19], Targetscan (https://www.targetscan.org/vert_80/, accessed on 9 April 2023, v8.0) [20], and starBase databases were used to predict the target genes of miRNAs. 

### 2.2. Clinical Tissue Samples

In this study, 30 paired LUAD and normal lung tissues were collected from our hospital (The Affiliated Cancer Hospital of Xiangya School of Medicine, Central South University/Hunan Cancer Hospital). The tissues were snap frozen in liquid nitrogen and stored until RNA extraction. All samples were identified by 2 senior pathologists. All patients signed an informed consent form, and this study was approved by the Ethics Committee of Hunan Cancer Hospital (Ethical lot number: KYJJ-2021-242). The clinical information of patients is reported in Table S1.

### 2.3. Cell Culture

LUAD cell lines (A549, H1975, and CALU-3) were cultured in RPMI 1640 medium (Gibco, Waltham, MA, USA), and human bronchial epithelioid cell line (HBE) and HEK293T cell lines were cultured in Dulbecco’s modified eagle medium (Gibco). The cell culture medium was supplemented with 10% fetal bovine serum (Gibco) and 1% penicillin/streptomycin (New Cell & Molecular Biotech, Suzhou, China). All cells were obtained from the cell bank of the Cancer Research Institute of Central South University (Changsha, China) [21,22] and incubated at 37 °C with 5% CO_2_. The cisplatin-resistant A549 cell line (A549/CDDP) was derived from A549 cells using stepwise increased cisplatin (Qilu Pharmaceutical, Shandong, China) concentration incubation. All cells were passaged to the third generation to start the experiments.

### 2.4. Plasmids, Mimics/Inhibitor, and Transfection

SATB2-overexpressing/shSATB2 plasmids and control plasmids were purchased from Vigene Biosciences (Rockville, MD, USA). The sequences of SATB2-3′UTR and *miR*-*660* were obtained from the National Center for Biotechnology Information database, and the target sequence of *miR*-*660* and SATB2 was predicted by the starBase database. The wild/mutant type of SATB2 (SATB2-WT/MUT) sequences are shown here: SATB2-WT, 5′-CCUAUCUGGCAGCUUAAUGGGUA-3′; SATB2-MUT, 5′-CCUAUCUGGCAGCUUUUACCCAU-3′. The SATB2-WT/MUT were synthesized from Vigene Biosciences. *miR*-*660* mimics/inhibitor and blank controls were obtained from Ribobio (Guangzhou, China). Transfection was carried out with Lipofectamine 3000 (Thermo Fisher Scientific, Waltham, MA, USA). 

The shRNA sequences are shown here: shSATB2-1, 5′-CCGGCTGCCGAAATTGACCAGAGATCTCGAGATCTCTGGTCAATTTCGGCAGTTTTTG-3′; shSATB2-2, 5′-CCGGGCCATGCAGAATTTCCTCAATCTCGAGATTGAGGAAATTCTGCATGGCTTTTTG-3′; shSATB2-3, 5′-CCGGGCCTTAAAGGAACTGCTCAAACTCGAGTTTGAGCAGTTCCTTTAAGGCTTTTTG-3′; shCTR, 5′-TTCTCCGAACGTGTCACGTTTCAAGAGAACGTGACACGTTCGGAGAATTTTTT-3′. 

### 2.5. RNA Isolation and Real-Time Quantitative Polymerase Chain Reaction (RT-qPCR) Assays 

The RNA sample was extracted using TRIzol solution (Thermo Fisher Scientific, Waltham, MA, USA). The first-strand complementary DNA was synthesized using a miDETECT A Track™ miRNA qRT-PCR Starter Kit (RiboBio) or RevertAid First Strand cDNA Synthesis Kit (Thermo Fisher Scientific), followed by PCR amplification in Bio-Rad CFX Connect™ Real-Time PCR Detection System (Bio-Rad, Hercules, CA, USA) or 2x SYBR Green qPCR Master Mix (Bimake, Houston, TX, USA) according to the manufacturer’s instructions. *U6* or *β*-*actin* was used as reference genes. Gene expression was analyzed using the comparative threshold cycle (2^−ΔΔCt^) method. 

The sequences of the primers (Tsingke, Beijing, China) used in this study are shown here: *β-actin*-F, 5′-CTCCATCCTGGCCTCGCTGT-3′; *β*-*actin*-R, 5′-GCTGTCACCTTCACCGTTCC-3′; *U6*-F, 5′-CTCGCTTCGGCAGCACA-3′, *U6*-R, 5′-AACGCTTCACGAATTTGCGT-3′; *SATB2*-F, 5′-CTTTGCAAGAGTGGCATTCA-3′; *SATB2*-R, 5′-GTTGTCGGTGTCGAGGTTTT-3′.

### 2.6. Cisplatin IC_50_ Detection

Cells (1000/well) were seeded into 96-well plates and incubated in 37 °C for 12 h. Then, the medium was replaced with a medium containing 0, 3, 12, 24, 36, and 48 μM cisplatin. After 48 h, Cell Counting Kit-8 (CCK-8, New Cell & Molecular Biotech) solution was added into the well, and the optical density values were measured at 450 nm (OD450). The relative cell viability (experimental group OD450/control group OD450×100%) with the corresponding cisplatin concentration was used to calculate the cell cisplatin IC_50_ values using nonlinear fitting analysis in GraphPad Prism 7 software (version 7.0, GraphPad, La Jolla, CA, USA).

### 2.7. Cell Proliferation

Cells (1000/well) were seeded into 96-well plates and incubated in 37 °C for 12 h. Then, the medium was replaced with a medium containing 10 μM cisplatin. The CCK-8 solution was added at the same time for 0, 1, 2, 3, 4, and 5 days, and the OD450 was measured. The results were visualized using GraphPad Prism 7 software. 

### 2.8. Colony Formation Assay

Cells (3000/well) were seeded into 6-well plates and cultured in medium containing 10 μM cisplatin for two weeks. The cells were fixed with methanol and stained with 0.1% crystal violet. The images were scanned, and the number of colonies was counted using Image J software (NIH Image J system, Bethesda, MD, USA, Version: 1.8.0).

### 2.9. Flow Cytometry

The cells were treated with 10 μM cisplatin for 48 h. Then, 2 × 10^5^ cells were collected and washed twice with phosphate-buffered saline. A Cell Cycle Staining Kit (Multi sciences, Hangzhou, China) was used to analyze the cell cycle, and Annexin V-FITC/propidium iodide (PI) Apoptosis Kit (Multi sciences) was used to perform the apoptosis assay according to the manufacturer’s instructions. The results were visualized using Modfit software (Verity Software House, Topsham, ME, USA, version LT3.1) or FlowJo software (BD Biosciences, Franklin Lakes, NJ, USA, version 10.0).

### 2.10. Luciferase Reporter Assay

HEK293T cells were co-transfected with *miR*-*660* mimics and SATB2-WT/MUT, together with Renilla luciferase pRLTK vector (Tsinke) as a control. After 24 h, the cells were lysed using radio immunoprecipitation assay buffer (RIPA, Beyotime, Shanghai, China). The Dual-Glo dual luciferase reporter assay system (Promega, Beijing, China) was utilized here to analyze and calculate the ratio of luminescence intensity. 

### 2.11. Western Blot

The proteins were extracted using RIPA (Beyotime) with a protease inhibitor (Beyotime). A BCA™ protein analysis kit (Thermo Fisher Scientific) was used to quantify the proteins. The SATB2 (Proteintech, Rosemont, IL, USA) was validated by Western blot analysis, and GAPDH (Proteintech) was used as a reference protein. HRP, Goat Anti-Rabbit IgG (Proteintech) was used as the secondary antibody. The signal was quantified using Image J software.

### 2.12. Statistical Analysis

All experiments were repeated at least three times. The data were analyzed using GraphPad Prism 7 software and were reported as the means ± standard deviation (SD). Student’s t-test and one-way analysis of variance were used to compare the two groups or multiple groups. Differences were considered significant when *p* < 0.05. * *p* < 0.05, ** *p* < 0.01, *** *p* < 0.001, and **** *p* < 0.0001.

## 3. Results

### 3.1. miR-660 Expression Is Associated with Cisplatin Sensitivity in LUAD Cells

**Figure 1:**
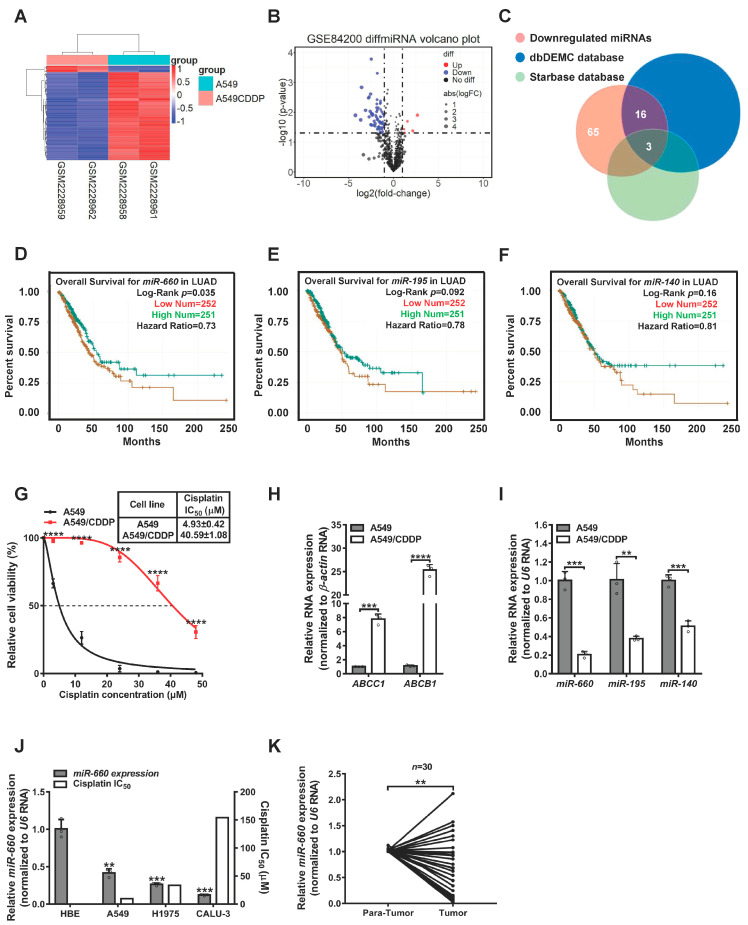
*MiR*-*660* expression is associated with cisplatin sensitivity in LUAD cells. (**A**,**B**) Heatmap and Volcano plot of the miRNA differential analysis using the GSE84200 dataset. (**C**) Venn diagram showing the intersection of miRNAs’ low expression in A549/CDDP cells, downregulated in tumor tissues with good prognosis in LUAD patients. (**D**–**F**) The relationship between *miR*-*660*, *miR*-*140,* and *miR*-*195* expression and prognosis of LUAD patients, according to the starbase database. (**G**) Cisplatin IC_50_ values of A549 and A549/CDDP cells. (**H**) The expression levels of two drug resistance markers, including *ABCC1* and *ABCB1*, in A549 and A549/CDDP cells were determined by RT-qPCR. (**I**) Expression levels of *miR*-*660*, *miR*-*140,* and *miR*-*195* in A549 and A549/CDDP cells measured by RT-qPCR. (**J**) Correlation of *miR*-*660* expression with cisplatin sensitivity in multiple LUAD cell lines, based on information in the GDSC database. (**K**) *miR*-*660* expression in LUAD tissues and paired adjacent nontumorous tissues (*n* = 30), measured by RT-qPCR. Data are presented as the mean ± standard deviation. ** *p* < 0.01, *** *p* < 0.001, **** *p* < 0.0001.

To identify the candidate miRNAs related to cisplatin resistance in LUAD, we reanalyzed the GSE84200 dataset, which contains miRNA expression profiling data of the A549 and A549/CDDP cell line. Then, we obtained 70 miRNAs with significant differences in expression, including 65 downregulated and 5 upregulated miRNAs in A549/CDDP cell line (Figure 1A,B). We queried the dbDEMC and starBase databases for the expression and prognosis of 65 of the above-mentioned miRNAs in LUAD. We found 16 miRNAs that were significantly downregulated in LUAD tumor tissues (Figure S1). In these 16 miRNAs, overexpression of *miR*-*660*, *miR*-*140,* and *miR*-*195* was closely related to better prognosis in LUAD patients (Figure 1C–F and Figure S2). 

We established the A549/CDDP cell line to verify the results of bioinformatics analysis. Cisplatin IC_50_ values variation (A549: 4.93 ± 0.42 μM, and A549/CDDP: 40.59 ± 1.08 μM) and drug resistance marker detection (ATP binding cassette subfamily c member 1, *ABCC1*; ATP binding cassette subfamily B member 1, *ABCB1*) [23,24] showed that the A549/CDDP cell line had been successfully established (Figure 1G,H). We found that all three above-mentioned miRNAs had lower expression levels in A549/CDDP cells and the lowest expression of *miR-660* was observed (Figure 1I). Compared with HBE cells, *miR*-*660* was stably downregulated in A549, H1975, and CALU-3 cells, while *miR*-*140* and *miR*-*195* were unstable (Figure 1J and Figure S3A). We obtained cisplatin IC_50_ values of these LUAD cell lines using the GDSC database (A549: 9.92 μM, H1975: 33.64 μM, and CALU-3: 154.02 μM) and found that the cisplatin IC_50_ values gradually enhanced with the *miR-660* expression stepwise decreasing (Figure 1J). Next, we examined *miR*-*660* expression in 30 paired LUAD tumor tissues and adjacent normal lung tissues and detected that *miR*-*660* expression was downregulated in most LUAD tissues (Figure 1K).

### 3.2. miR-660 Enhances Cisplatin Sensitivity in LUAD Cells

**Figure 2:**
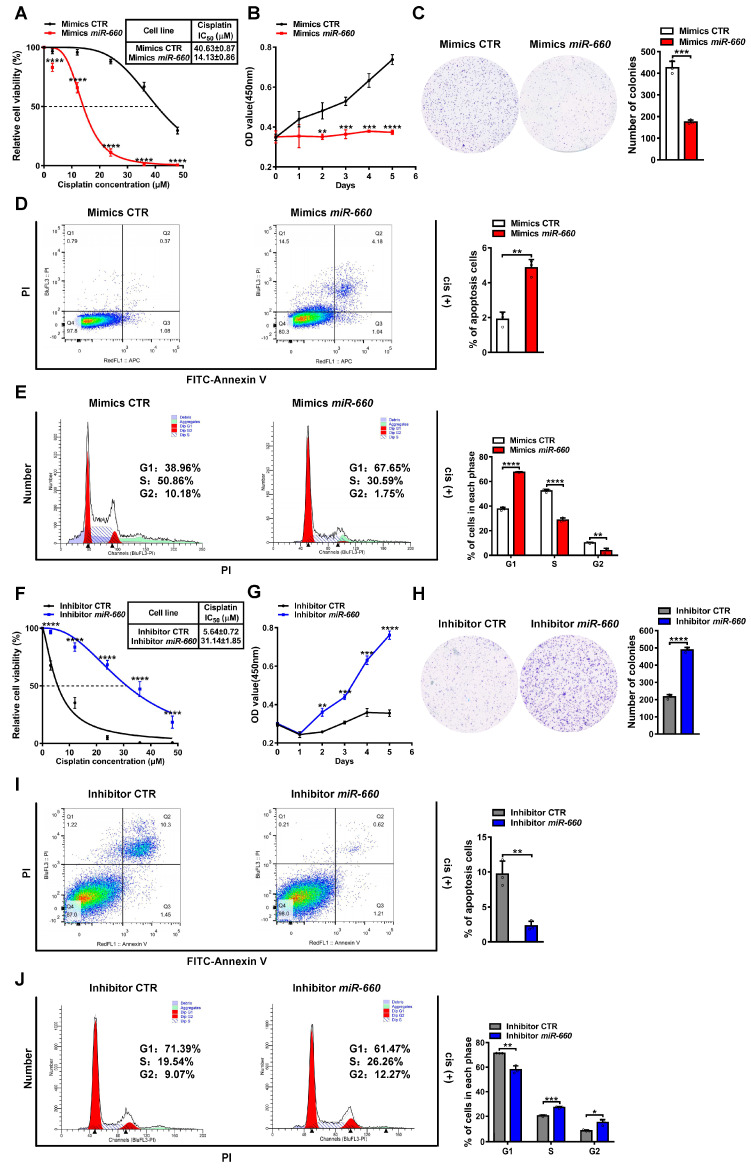
*MiR*-*660* enhances cisplatin sensitivity in LUAD cells. (**A**) Comparison of cisplatin IC_50_ values between A549/CDDP cells transfected with mimics of CTR and *miR*-*660* mimics. (**B**–**E**) In cisplatin-treated A549/CDDP cells after transfection with *miR*-*660* mimics and mimics of CTR, the capacities of cell proliferation and colony formation were examined using CCK8 and colony formation assays. The apoptosis rate and cell cycle detection were determined by flow cytometry assays. (**F**) Comparison of cisplatin IC_50_ values between A549 cells transfected with inhibitor CTR and *miR*-*660* inhibitor. (**G**–**J**) In cisplatin-treated A549 cells after transfection with *miR*-*660* inhibitor and inhibitor CTR, the capacities of cell proliferation and colony formation were examined using CCK8 and colony formation assays. The apoptosis rate and cell cycle detection were determined by flow cytometry assays. Data are presented as the mean ± standard deviation. * *p* < 0.05, ** *p* < 0.01, *** *p* < 0.001, **** *p* < 0.0001.

In order to investigate the function of *miR*-*660* in LUAD cisplatin resistance, the expression of *miR*-*660* was upregulated using mimics in A549/CDDP cells and CALU-3 cells and knocked down using inhibitors in A549 cells, respectively (Figure S3B). Subsequently, a series of experiments was executed in vitro. The cisplatin IC_50_ detection showed that *miR*-*660* overexpression decreased the cisplatin IC_50_ value from 40.63 ± 0.87 μM to 14.13 ± 0.86 μM in A549/CDDP cells (Figure 2A). *MiR*-*660* upregulation also decreased the proliferation and colony formation capacity in A549/CDDP cells treated with cisplatin. Meanwhile, the cell apoptosis rate and G1 cell cycle arrest increased, and S-G2 cell cycle arrest decreased in cisplatin-treated A549/CDDP cells (Figure 2B–E). In contrast, cisplatin IC_50_ detection showed that *miR*-*660* downregulation increased cisplatin resistance in A549 cells (Figure 2F). The *miR*-*660* inhibitor notably increased the proliferation and colony formation capacity in A549 cells with cisplatin treatment. Meanwhile, the cell apoptosis rate and G1 cell cycle arrest decreased, and S-G2 cell cycle arrest increased in cisplatin-treated A549 cells (Figure 2G–J). In addition, *miR*-*660* overexpression induced a decrease in cell proliferation and an decrease in G2 cell cycle arrest in cisplatin-treated CALU-3 cells (Figure S3C–F). Taken together, these results showed that *miR*-*660* could efficiently increase cisplatin sensitivity in LUAD cells.

### 3.3. SATB2 Is a Direct Target of miR-660

**Figure 3:**
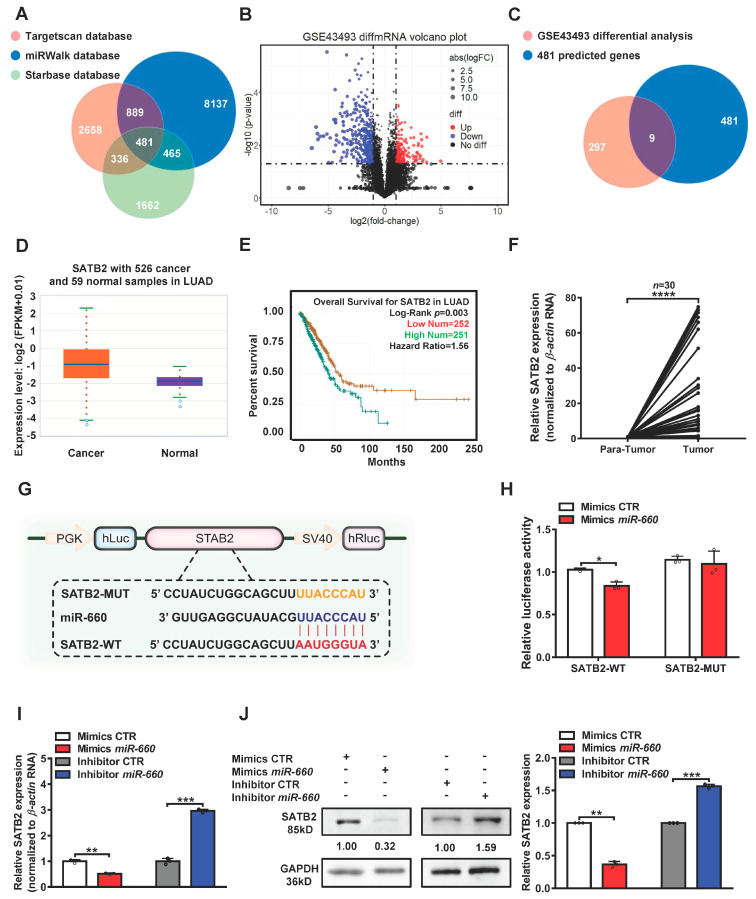
SATB2 is a direct target of *miR*-*660*. (**A**) Venn diagram of the results of three miRNA databases predicting *miR*-*660* target genes. (**B**) Volcano plot of the mRNA differential analysis using the GSE43493 dataset. (**C**) Venn diagram showing the intersection of 481 predicted target genes of *miR*-*660* and 297 upregulated genes in A549/CDDP cells. (**D**,**E**) The expression level of SATB2 in LUAD tissue and the relationship between SATB2 expression and prognosis of LUAD patients, according to the starbase database. (**F**) SATB2 expression in LUAD tissues and paired adjacent nontumorous tissues (*n* = 30), measured by RT-qPCR. (**G**) Pattern diagram of the target sequences of *miR*-*660* and SATB2. (**H**) Double luciferase report assay to verify the binding of *miR*-*660* and SATB2. (**I**,**J**) The expression relationship between *miR*-*660* and SATB2 was determined by RT-qPCR and Western blot. Data are presented as mean ± standard deviation. * *p* < 0.05, ** *p* < 0.01, *** *p* < 0.001, **** *p* < 0.0001.

Using three target-prediction databases (miRWalk, Targetscan, and starBase), 481 predicted target genes of *miR*-*660* were identified (Figure 3A). Comparing differential genes from the GSE43493 dataset, which contains mRNA expression profiling data of the A549 and A549/CDDP cell lines, we found 297 upregulated genes in the A549/CDDP group compared with the A549 group (Figure 3B). Interestingly, there were 9 potential *miR-660* downstream target genes also upregulated in the A549/CDDP cell line, namely PTP4A1, HAS3, LFNG, ANKS1A, BCL2, GAS7, SATB2, FAM173B, and MKRN1 (Figure 3C). We examined the expression and prognosis of these target genes in the starBase database and found that SATB2 was upregulated in LUAD tissues and overexpression of SATB2 was related to poor prognosis in patients with LUAD (Figure 3D,E and Figure S4). To verify the results of the bioinformatics analysis, we detected the expression of SATB2 in paired LUAD and normal lung tissues using RT-qPCR assay. We found that SATB2 was indeed overexpressed in most tumor tissues (Figure 3F). We obtained the target sequences of *miR*-*660* and SATB2. We found that *miR*-*660* overexpression repressed the luciferase activity of reporter plasmids containing SATB2-WT in HEK293T cells, while the luciferase activity of SATB2-MUT plasmids was not altered (Figure 3G,H). In A549/CDDP cells, upregulated *miR*-*660* led to an obvious decrease in SATB2 expression. Conversely, suppressing *miR*-*660* resulted in upregulation of SATB2 expression in A549 cells (Figure 3I,J and Figure S5A). These results suggest that SATB2 could be a direct target of *miR*-*660* in LUAD cells. 

### 3.4. miR-660 Regulates Cisplatin Sensitivity in LUAD Cells through SATB2

**Figure 4:**
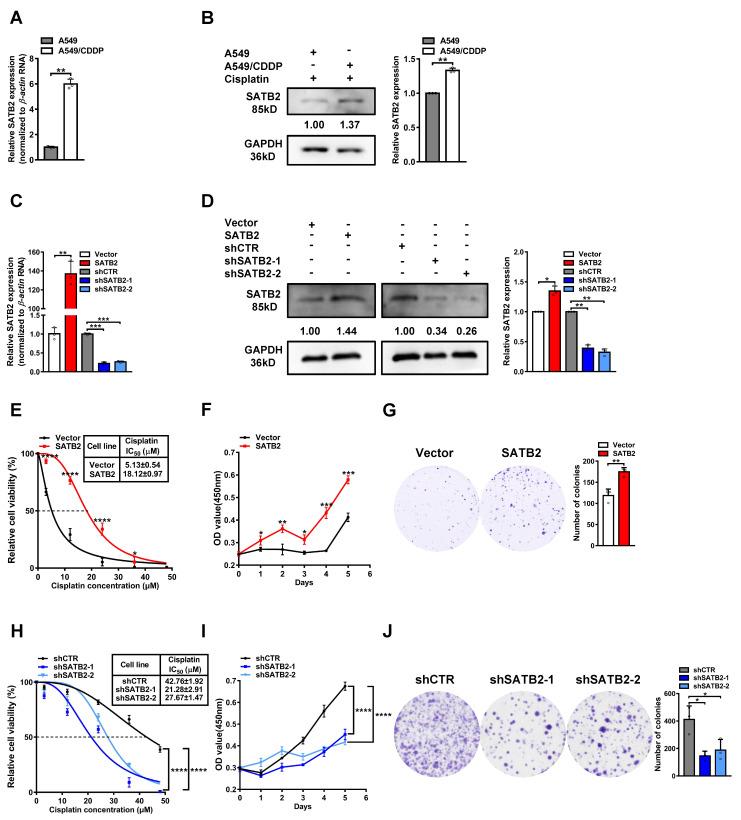
SATB2 promotes cisplatin resistance in LUAD cells. (**A**,**B**) Expression levels of SATB2 in A549 and A549/CDDP cells were measured by RT-qPCR and Western blot. (**C**,**D**) The effect of SATB2 overexpression and knockdown were determined by RT-qPCR and Western blot. (**E**) Comparison of cisplatin IC_50_ values between the A549-Vector and A549-SATB2 cells. (**F**,**G**) With cisplatin treatment, the capacities of cell proliferation and colony formation in A549-SATB2 cells and the corresponding control cells were examined using CCK8 and colony formation assays. (**H**) Comparison of cisplatin IC_50_ values between A549/CDDP-shCTR and A549/CDDP-shSATB2 cells. (**I**,**J**) With cisplatin treatment, the capacities of cell proliferation and colony formation in A549/CDDP-shSATB2 cells and the corresponding control cells were examined using CCK8 and colony formation assays. Data are presented as the mean ± standard deviation. * *p* < 0.05, ** *p* < 0.01, *** *p* < 0.001, **** *p* < 0.0001.

We detected SATB2 expression in A549 and A549/CDDP cells. After cisplatin treatment, compared with A549 cells, the results of RT-qPCR and Western blot showed that SATB2 was upregulated in A549/CDDP cells (Figure 4A,B and Figure S5B). To observe the function of SATB2 in LUAD cisplatin resistance, we first transfected SATB2 overexpression plasmid into A549 cells and shSATB2 into A549/CDDP cells (Figure 4C,D and Figure S5C). The cisplatin IC_50_ detection showed that SATB2 overexpression increased cisplatin resistance and promoted cell proliferation in A549 cells (Figure 4E–G). In contrast, cisplatin IC_50_ detection showed that SATB2 knockdown decreased cisplatin resistance and inhibited cell proliferation in A549/CDDP cells (Figure 4H–J). These results proved that SATB2 could indeed promote cisplatin resistance in LUAD cells. 

**Figure 5:**
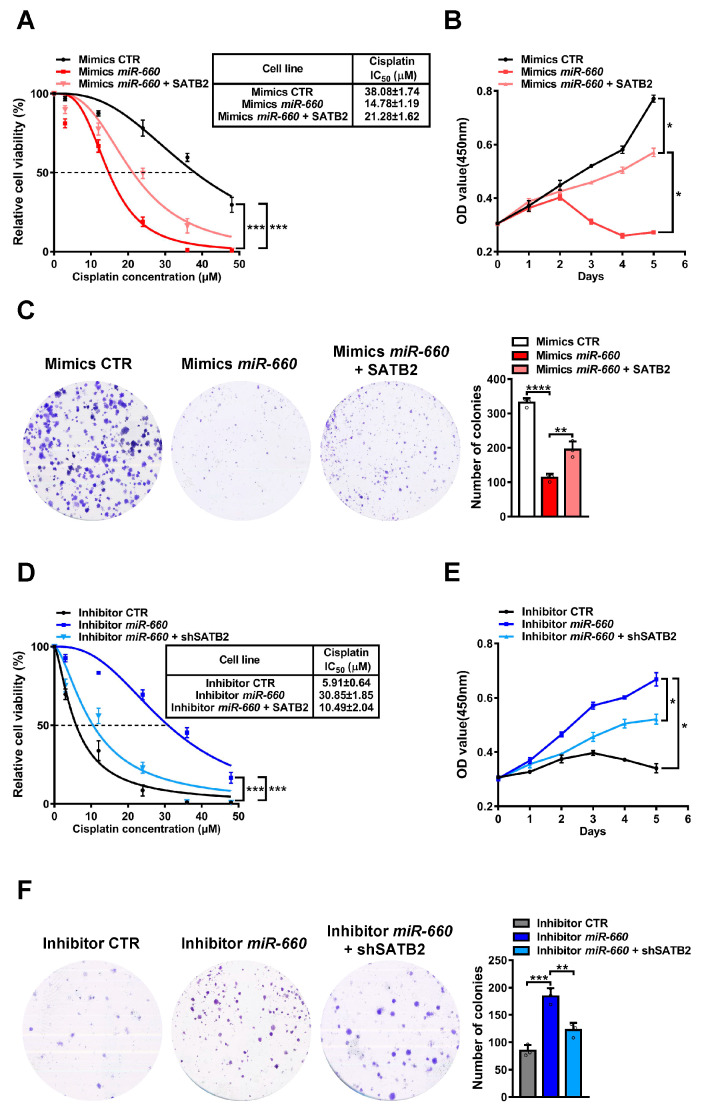
*MiR*-*660* regulates cisplatin sensitivity in LUAD cells through SATB2. (**A**) Comparison of cisplatin IC_50_ values between mimics CTR, *miR*-*660* mimics, and *miR*-*660* mimics + SATB2 in A549/CDDP cells. (**B**,**C**) With cisplatin treatment, the capacities of cell proliferation and colony formation of A549/CDDP cells in mimics CTR, *miR*-*660* mimics, and *miR-660* mimics + SATB2 groups were examined using CCK8 and colony formation assays. (**D**) Comparison of cisplatin IC_50_ values between inhibitor CTR, *miR*-*660* inhibitor, and *miR*-*660* inhibitor + shSATB2 in A549 cells. (**E**,**F**) With cisplatin treatment, the capacities of cell proliferation and colony formation of A549 cells in inhibitor CTR, *miR*-*660* inhibitor, and *miR*-*660* inhibitor + shSATB2 groups were examined using CCK8 and colony formation assays. Data are presented as the mean ± standard deviation. * *p* < 0.05, ** *p* < 0.01, *** *p* < 0.001, **** *p* < 0.0001.

Finally, we conducted rescue experiments to verify whether *miR*-*660* relied on SATB2 to influence cisplatin resistance in LUAD. In cisplatin-treated A549/CDDP cells, the decrease in cisplatin IC_50_ value and proliferation capacity caused by *miR*-*660* upregulation was completely rescued by SATB2 overexpression (Figure 5A–C). Moreover, co-transfection of *miR*-*660* inhibitor and shSATB2 plasmid in cisplatin-treated A549 cells weakened the enhanced cisplatin resistance brought by *miR*-*660* knockdown (Figure 5D–F). Taken together, we clarified that *miR-660* inhibits LUAD cisplatin resistance by decreasing SATB2 expression.

## 4. Discussion

To obtain candidate miRNAs related to cisplatin resistance in LUAD, we analyzed the LUAD cisplatin-resistant/sensitive cell dataset GSE84200 and obtained 65 miRNAs with downregulation in LUAD cisplatin-resistant cells. After successful establishment of the A549/CDDP cell line, we found that *miR*-*660* was not only lowly expressed in LUAD cells and tissues but also further decreased in the LUAD cisplatin-resistant cell line. In addition, *miR*-*660* downregulation was associated with poor prognosis in LUAD patients.

*miR*-*660* was upregulated in breast and liver cancer [25,26,27,28]. *miR*-*660* had low expression in lung cancer and targeted MDM2, which induced destabilization of the p53 protein, thus promoting cell proliferation in lung cancer [29]. *miR*-*660* could inhibit lung cancer progression and bone metastasis by regulating SMARCA5 expression [30]. *CircFARP1* increased leukemia inhibitory factor (LIF) expression through sponging *miR*-*660,* which enhanced gemcitabine resistance in pancreatic ductal adenocarcinoma [31]. Until now, there has been no research report on the relationship between *miR*-*660* and cisplatin resistance in LUAD. Cisplatin promotes tumor cell apoptosis by forming an adduct with the DNA of tumor cells, thereby achieving its anti-tumor effect [32]. Many studies have confirmed that the decrease in cell cycle arrest is the key factor in tumor cisplatin resistance [33,34,35]. We found that the overexpression of *miR*-*660* decreased the cell proliferation capacity and increased the cell apoptosis rate and cell cycle arrest in cisplatin-treated A549/CDDP and CALU-3 cells. These results suggest that *miR*-*660* may be a key regulator in LUAD cisplatin sensitivity.

To identify the potential mechanism of *miR*-*660* in cisplatin resistance, we used multiple miRNA databases and analyzed upregulated mRNAs in cisplatin resistance A549 cells. We found that *miR*-*660*-targeted SATB2 was overexpressed in LUAD tissues and A549/CDDP cells, and the prognosis of LUAD patients with SATB2 overexpression was poor. SATB2 was found to be overexpressed in a variety of cancers, including colorectal cancer, hepatocellular carcinoma, and neuroendocrine tumors, and played an important role in the occurrence, development, and metastasis of cancers [36,37,38,39,40,41,42,43,44]. In head and neck cancer, SATB2 could bind with ΔNp63a to inhibit downstream Tap73β transcription which enhanced chemotherapy resistance of cancer cells [45]. In our research, a dual-luciferase reporter assay confirmed the combination of *miR*-*660* and SATB2. RT-qPCR and Western blot simultaneously proved that the expression of *miR*-*660* and SATB2 was negatively correlated. After cisplatin treatment, we found that SATB2 was overexpressed in A549/CDDP cells and SATB2 knockdown decreased the proliferation ability of A549/CDDP cells. SATB2 overexpression enhanced cisplatin resistance in A549 cells. Moreover, rescue experiments verified that *miR*-*660* regulated cisplatin sensitivity in LUAD cells through SATB2. The expression regulation mechanism of *miR*-*660* in A549/CDDP cells and how SATB2 regulated cisplatin resistance in LUAD will be studied in future studies.

In this study, we found that the downregulation of *miR*-*660* increased the cisplatin resistance of LUAD cells by enhancing the expression of SATB2. *miR*-*660*/SATB2 axis may be a key regulator of cisplatin treatment effect in LUAD.

## Data Availability

Data used or analyzed during the current study are available from the corresponding author upon reasonable request.

## Supplementary Materials

The following supporting information can be downloaded at: https://www.mdpi.com/article/10.3390/genes14040911/s1, Table S1 Clinical information of 30 LUAD patients. Figure S1 Expression of 16 candidate miRNAs in multiple tumors, according to the dbDEMC database. Figure S2 The relationship between the expression of 13 remaining candidate miRNAs and prognosis of LUAD patients, according to the starbase database. Figure S3A The expression of *miR*-*195* and *miR*-*140* in LUAD cells was detected using RT-qPCR. Figure S3B The effects of *miR-660* mimics and inhibitors were determined by RT-qPCR. Figure S3C–F With cisplatin treatment, the capacities of cell proliferation and colony formation in CALU-3 mimics *miR*-*660* cells and the corresponding control cells were examined using CCK8 and colony formation assays. The apoptosis rate and cell cycle detection were determined by flow cytometry assays. Data are presented as the mean ± standard deviation. * *p* < 0.05, ** *p* < 0.01, *** *p* < 0.001, **** *p* < 0.0001. Figure S4 The expression and survival analysis results of the remaining eight *miR*-*660* potential target genes, according to the starbase database. Figure S5A The expression relationship between *miR*-*660* and SATB2 was determined by Western blot. Figure S5B Expression levels of SATB2 in A549 and A549/CDDP cells were measured by Western blot. Figure S5C The effects of SATB2 overexpression and knockdown were determined by Western blot. Data are presented as the mean ± standard deviation. * *p* < 0.05, ** *p* < 0.01, *** *p* < 0.001.
