# Supplementary material for: microRNA-660 Enhances Cisplatin Sensitivity via Decreasing SATB2 Expression in Lung Adenocarcinoma
Source: Genes (Basel). 2023 Apr 14;14(4):911. doi: 10.3390/genes14040911 (PMC10137726; doi:10.3390/genes14040911)

Differential Expression Profile in cancer vs normal

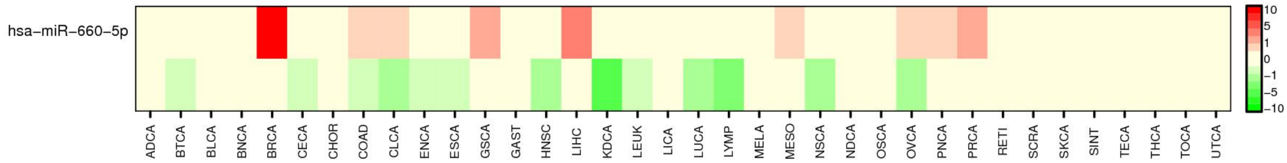

Differential Expression Profile in cancer vs normal

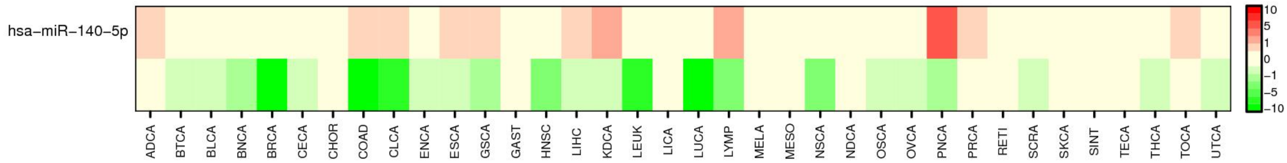

Differential Expression Profile in cancer vs normal

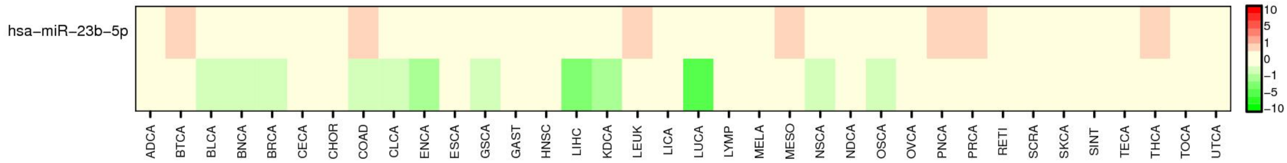

Differential Expression Profile in cancer vs normal

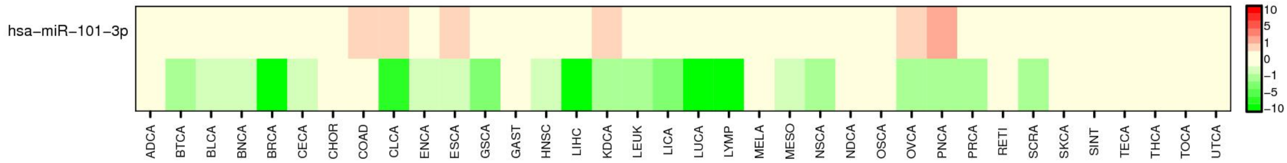

Differential Expression Profile in cancer vs normal

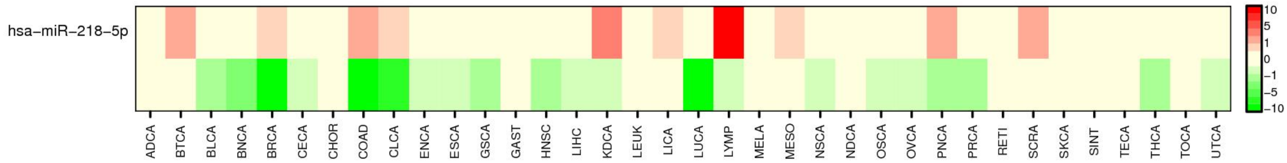

Differential Expression Profile in cancer vs normal

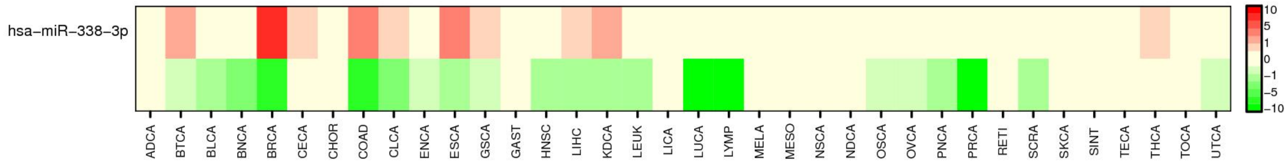

Differential Expression Profile in cancer vs normal

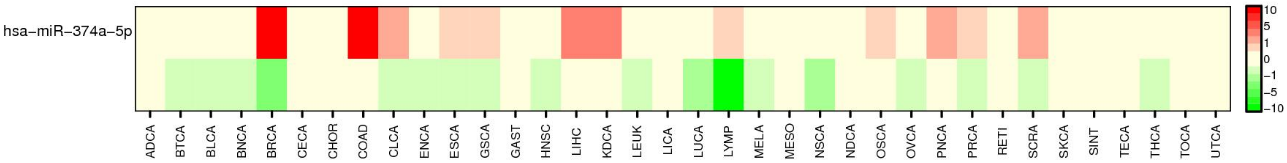

Differential Expression Profile in cancer vs normal

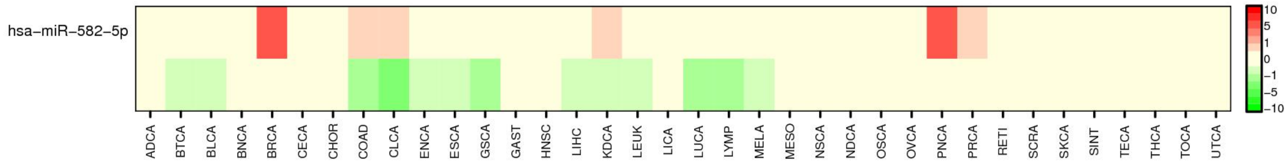

Differential Expression Profile in cancer vs normal

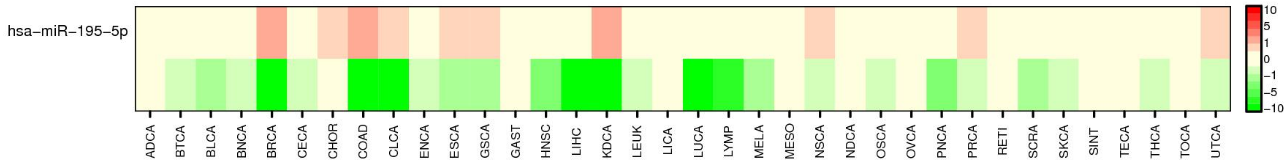

Differential Expression Profile in cancer vs normal

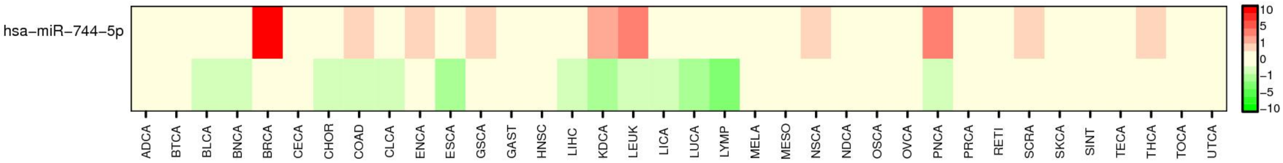

Differential Expression Profile in cancer vs normal

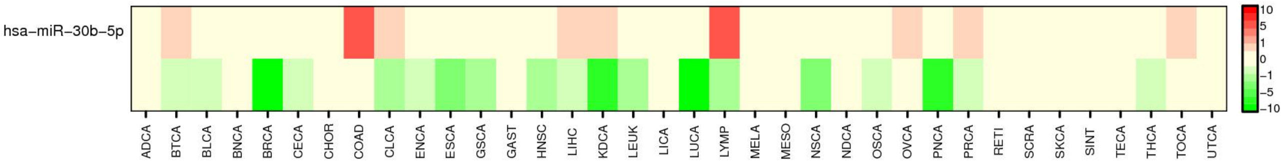

Differential Expression Profile in cancer vs normal

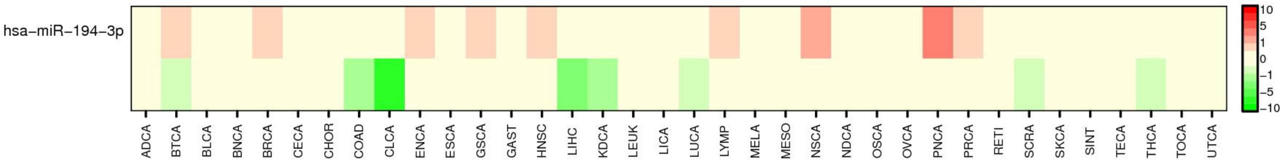

Differential Expression Profile in cancer vs normal

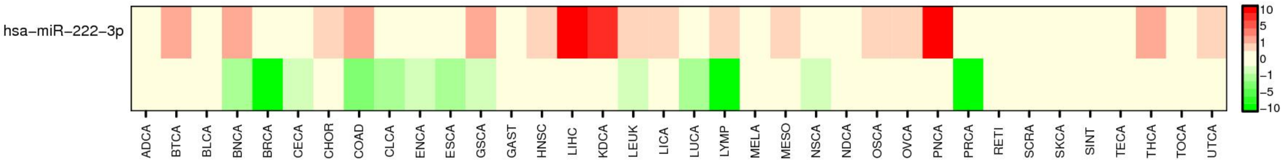

Differential Expression Profile in cancer vs normal

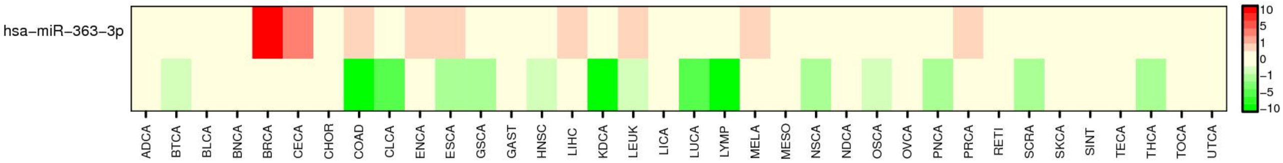

Differential Expression Profile in cancer vs normal

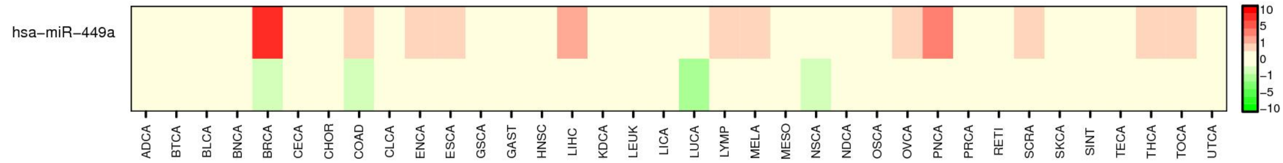

Differential Expression Profile in cancer vs normal

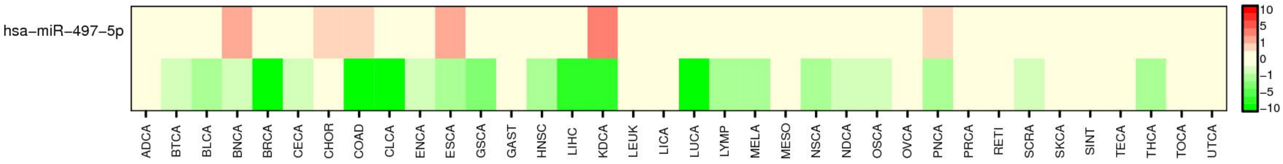

Supplement: Supplementary file 1 [file genes-14-00911-s001.zip › Fig. S1.pdf]
