# Supplementary material for: microRNA-660 Enhances Cisplatin Sensitivity via Decreasing SATB2 Expression in Lung Adenocarcinoma
Source: Genes (Basel). 2023 Apr 14;14(4):911. doi: 10.3390/genes14040911 (PMC10137726; doi:10.3390/genes14040911)
Supplement: Supplementary file 1 [file genes-14-00911-s001.zip › Table S1.pdf]

Table S1 Clinical information of 30 LUAD patients.

Number	Age	Sex	Pathology diagnosis	TX	NX	MX
1	49	male	moderately-poorly differentiated lung adenocarcinoma	2	1	0
2	51	male	well-moderately differentiated lung adenocarcinoma	2	0	0
3	55	female	moderately differentiated lung adenocarcinoma	2	0	0
4	58	male	moderately-poorly differentiated lung adenocarcinoma	4	0	0
5	53	female	moderately-poorly differentiated lung adenocarcinoma	4	0	0
6	66	female	moderately-poorly differentiated lung adenocarcinoma	3	2	0
7	73	male	moderately differentiated lung adenocarcinoma	1	0	0
8	67	female	well-moderately differentiated lung adenocarcinoma	2	0	0
9	58	female	moderately-poorly differentiated lung adenocarcinoma	3	1	0
10	63	female	well-moderately differentiated lung adenocarcinoma	1	0	0
11	75	male	moderately differentiated lung adenocarcinoma	2	1	0
12	79	male	moderately differentiated lung adenocarcinoma	2	0	0
13	65	male	moderately differentiated lung adenocarcinoma	1	0	0
14	66	male	moderately-poorly differentiated lung adenocarcinoma	4	0	0
15	53	male	moderately differentiated lung adenocarcinoma	2	0	0
16	55	male	moderately-poorly differentiated lung adenocarcinoma	3	1	0
17	59	female	moderately differentiated lung adenocarcinoma	1	0	0
18	55	male	well-moderately differentiated lung adenocarcinoma	1	0	0
19	61	female	moderately-poorly differentiated lung adenocarcinoma	3	1	0
20	63	female	moderately-poorly differentiated lung adenocarcinoma	4	0	0
21	76	male	moderately-poorly differentiated lung adenocarcinoma	2	1	0

22	70	female	moderately differentiated lung adenocarcinoma	1	0	0
23	58	male	well-moderately differentiated lung adenocarcinoma	1	0	0
24	59	female	moderately differentiated lung adenocarcinoma	2	1	0
25	59	female	well-moderately differentiated lung adenocarcinoma	2	0	0
26	61	female	well-moderately differentiated lung adenocarcinoma	1	0	0
27	69	male	well-moderately differentiated lung adenocarcinoma	2	0	0
28	73	male	moderately differentiated lung adenocarcinoma	1	0	0
29	76	male	moderately-poorly differentiated lung adenocarcinoma	3	1	0
30	66	female	well-moderately differentiated lung adenocarcinoma	1	0	0
