# Supplementary figures and images for: microRNA-660 Enhances Cisplatin Sensitivity via Decreasing SATB2 Expression in Lung Adenocarcinoma
Source: Genes (Basel). 2023 Apr 14;14(4):911. doi: 10.3390/genes14040911 (PMC10137726; doi:10.3390/genes14040911)

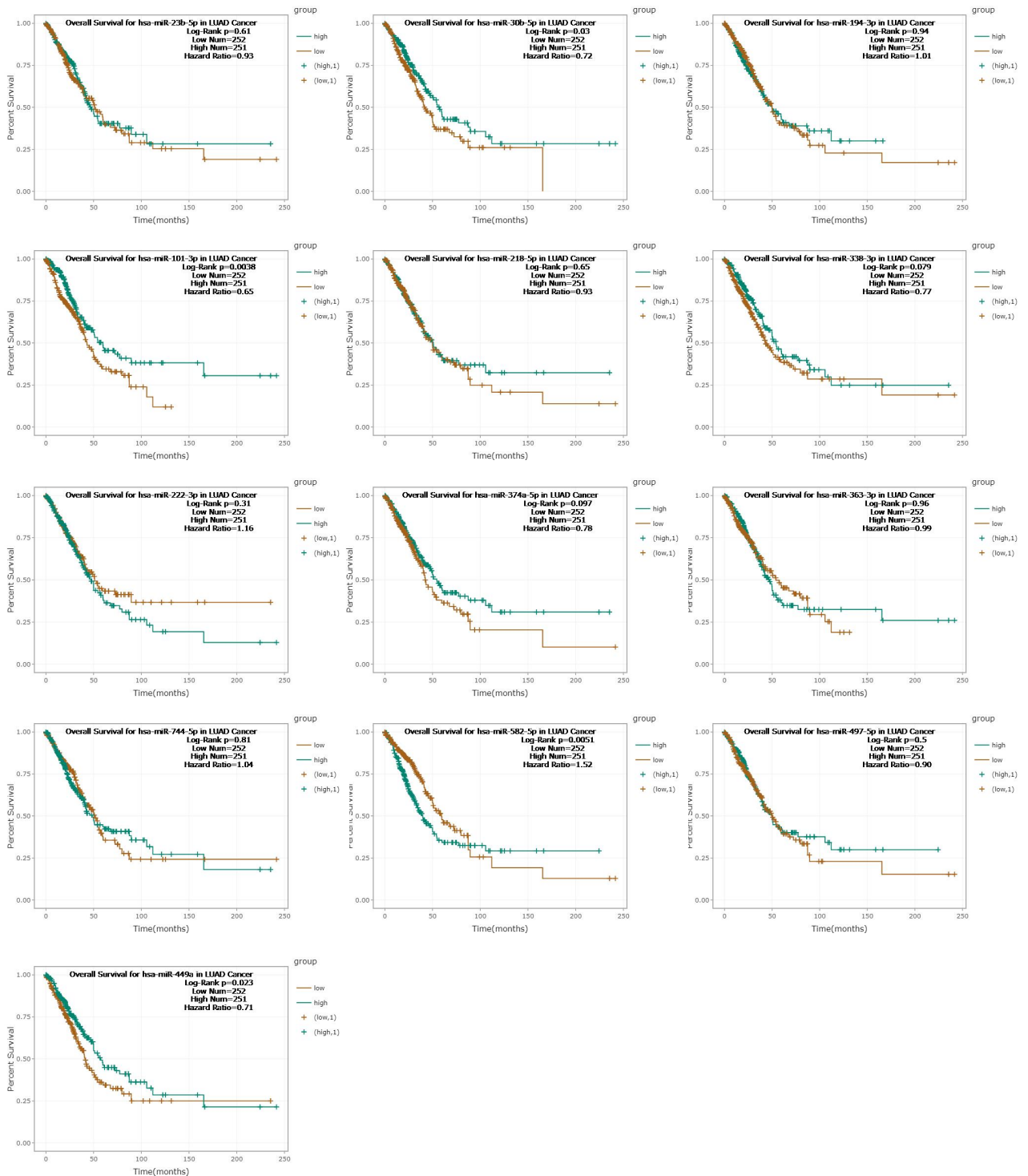

Supplement: Supplementary file 1 [file genes-14-00911-s001.zip › Fig. S2.pdf]

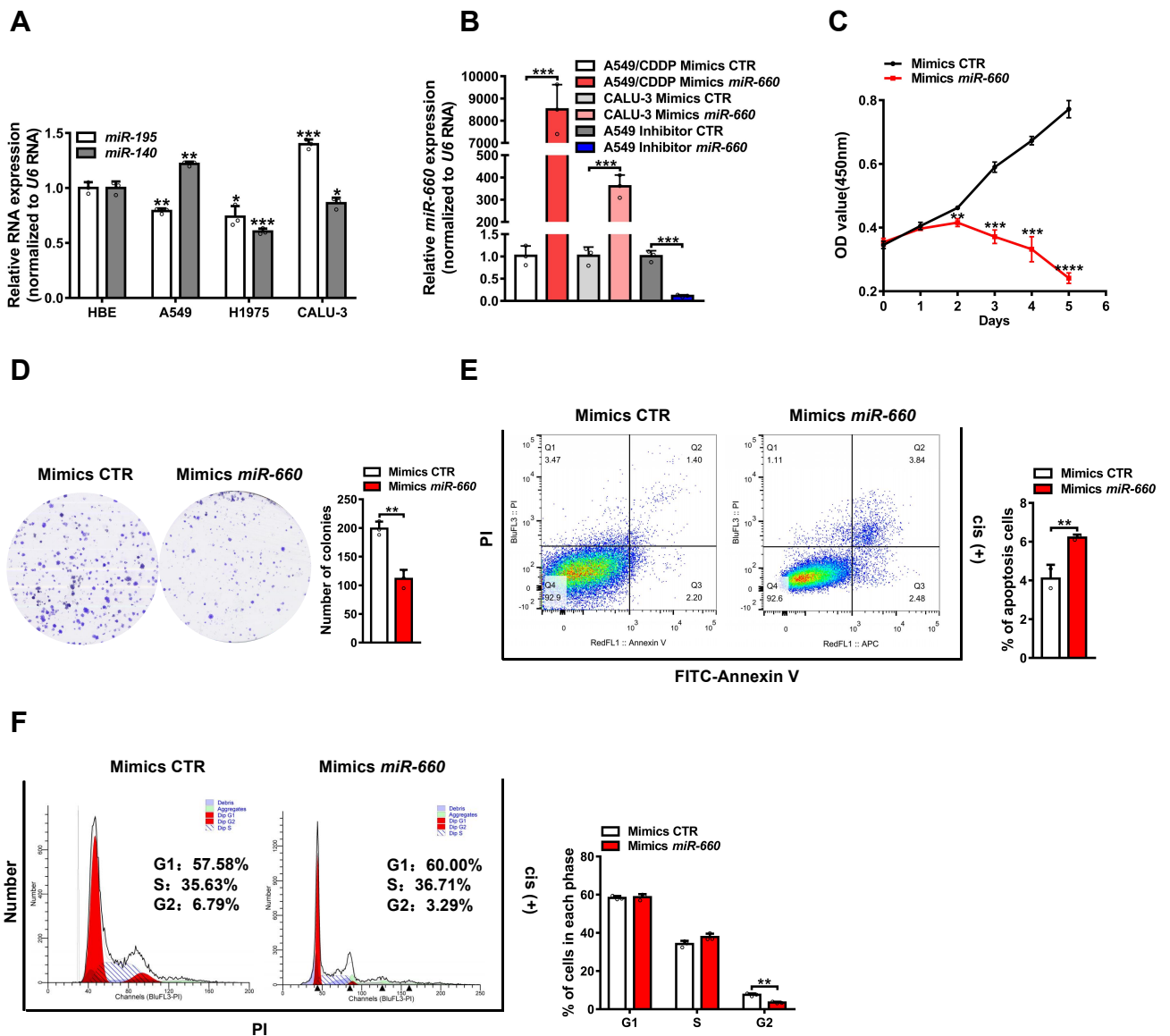

Supplement: Supplementary file 1 [file genes-14-00911-s001.zip › Fig. S3.pdf]

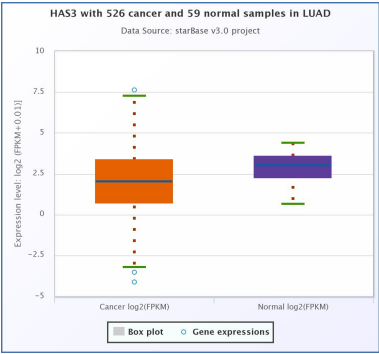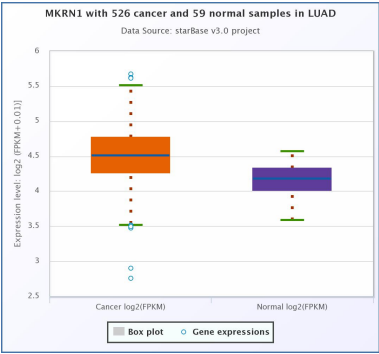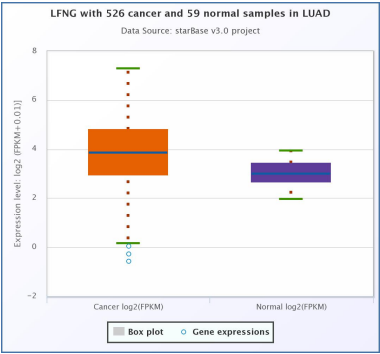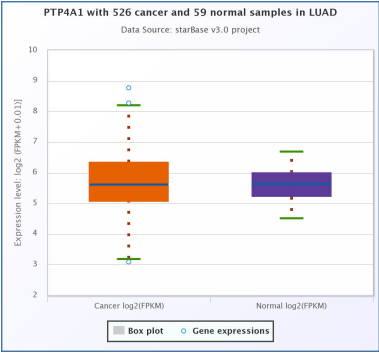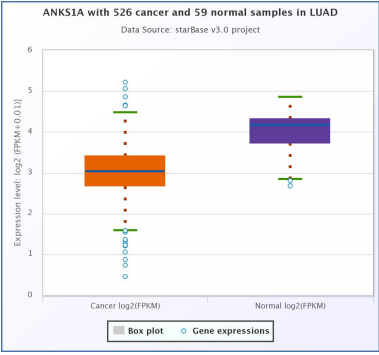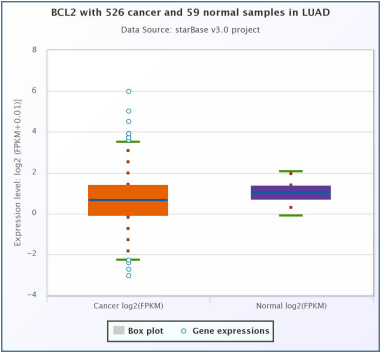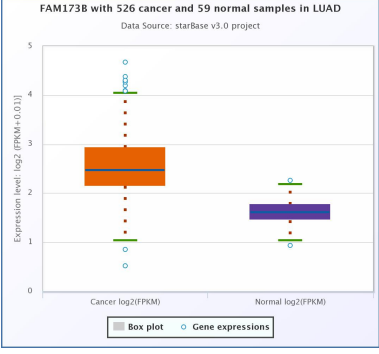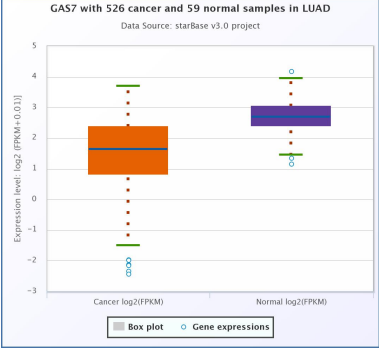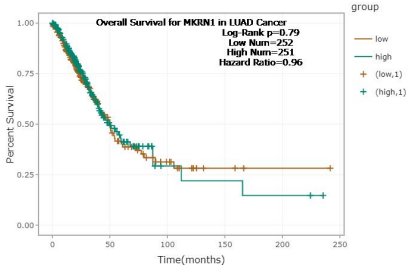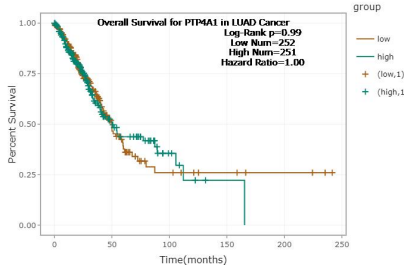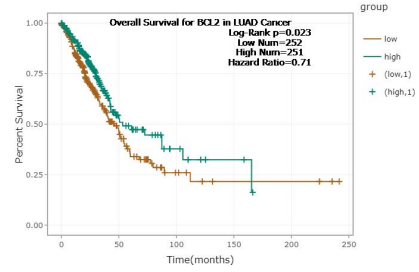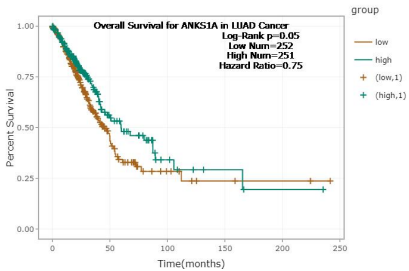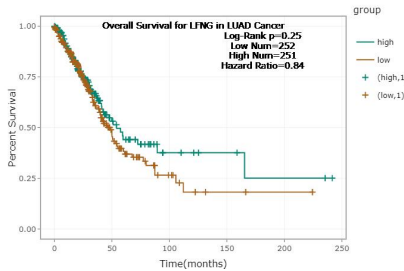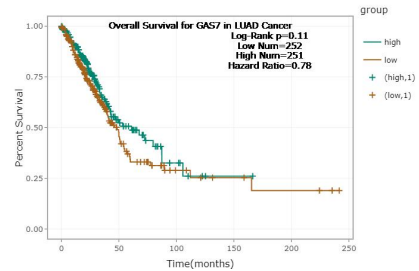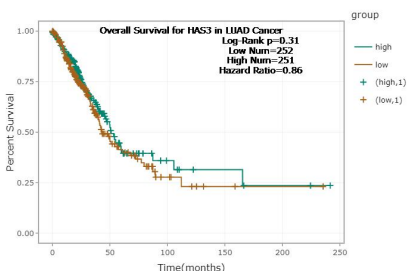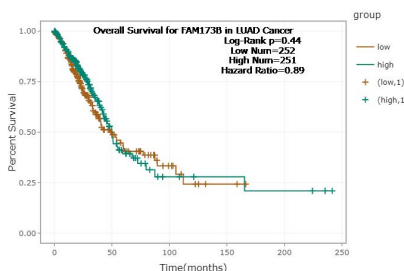

Supplement: Supplementary file 1 [file genes-14-00911-s001.zip › Fig. S4.pdf]

# A

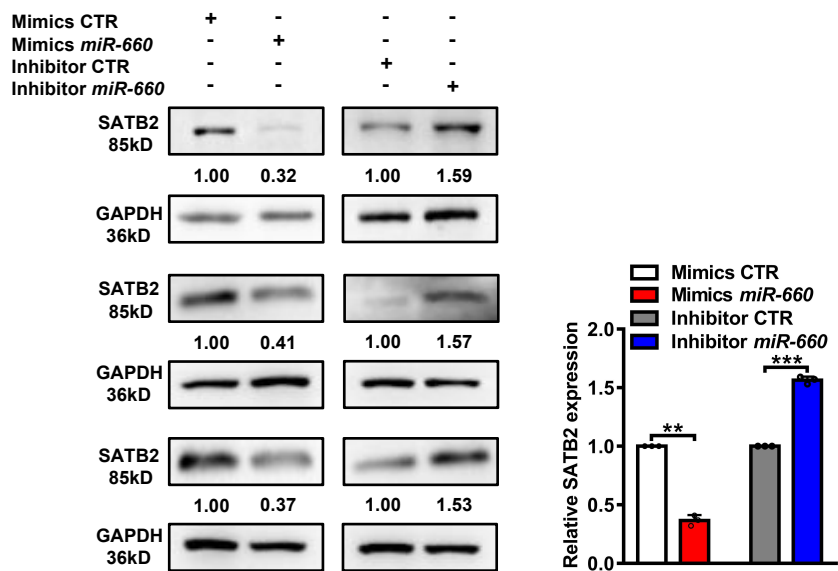

# B

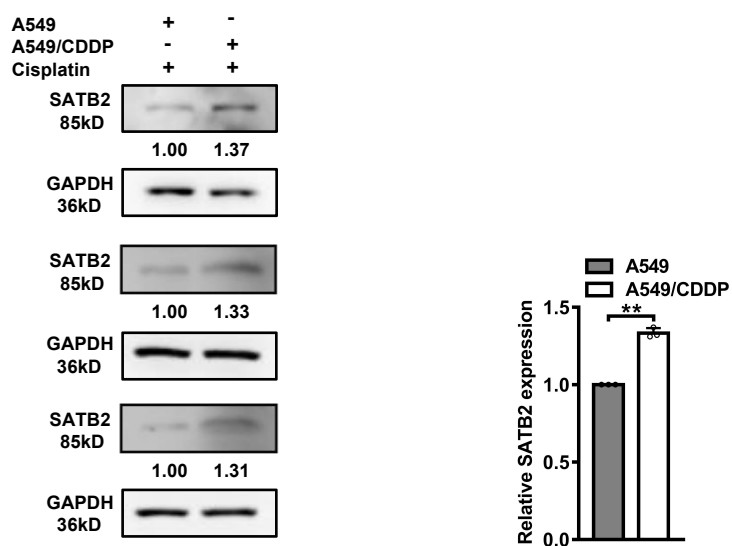

# C

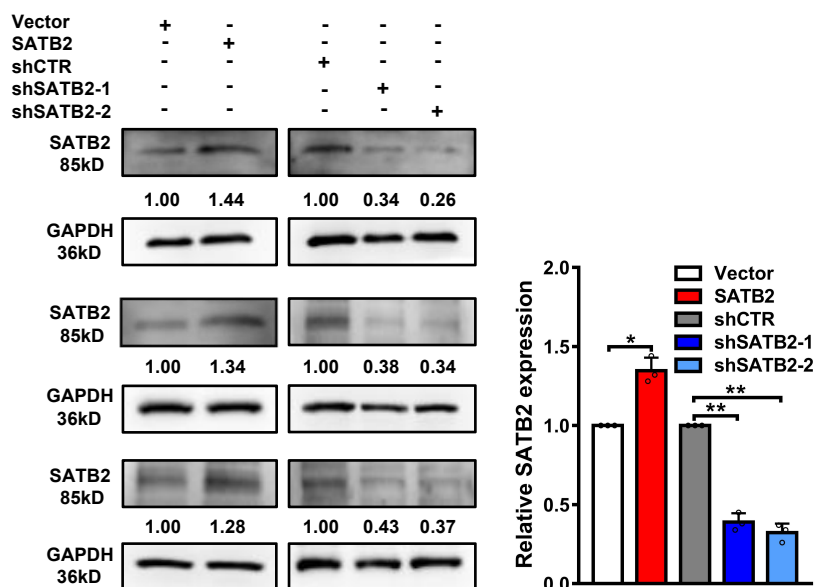

Supplement: Supplementary file 1 [file genes-14-00911-s001.zip › Fig. S5.pdf]
